# Cortical and Subcortical Anatomy of the Parietal Lobe From the Neurosurgical Perspective

**DOI:** 10.3389/fneur.2021.727055

**Published:** 2021-08-26

**Authors:** Tomasz Andrzej Dziedzic, Aleksandra Bala, Andrzej Marchel

**Affiliations:** ^1^Department of Neurosurgery, Medical University of Warsaw, Warsaw, Poland; ^2^Faculty of Psychology, University of Warsaw, Warsaw, Poland

**Keywords:** white matter, anatomy, fiber dissection, tractography, glioma, parietal lobe

## Abstract

**Introduction:** The anatomical structures of the parietal lobe at the cortical and subcortical levels are related mainly to sensory, visuospatial, visual and language function. The aim of this study was to present an intraoperative perspective of these critical structures in terms of the surgical treatment of intra-axial lesions. The study also discusses the results of the technique and the results of direct brain stimulation under awake conditions.

**Materials and Methods:** Five adult brains were prepared according to the Klingler technique. Cortical assessments and all measurements were performed with the naked eye, while white matter dissection was performed with microscopic magnification.

**Results:** Intra-axial lesions within the parietal lobe can be approached through a lateral or superior trajectory. This decision is based on the location of the lesions in relation to the arcuate fascicle/superior longitudinal fascicle (AF/SLF) complex and ventricular system. Regardless of the approach, the functional borders of the resection are defined by the postcentral gyrus anteriorly and Wernicke's speech area inferiorly. On the subcortical level, active identification of the AF/SLF complex and of the optic radiation within the sagittal stratum should be performed. The intraparietal sulcus (IPS) is a reliable landmark for the AF/SLF complex in ~60% of cases.

**Conclusion:** Knowledge of the cortical and subcortical anatomical and functional borders of the resection is crucial in preoperative planning, prediction of the risk of postoperative deficits, and intraoperative decision making.

## Introduction

**Figure 1:**
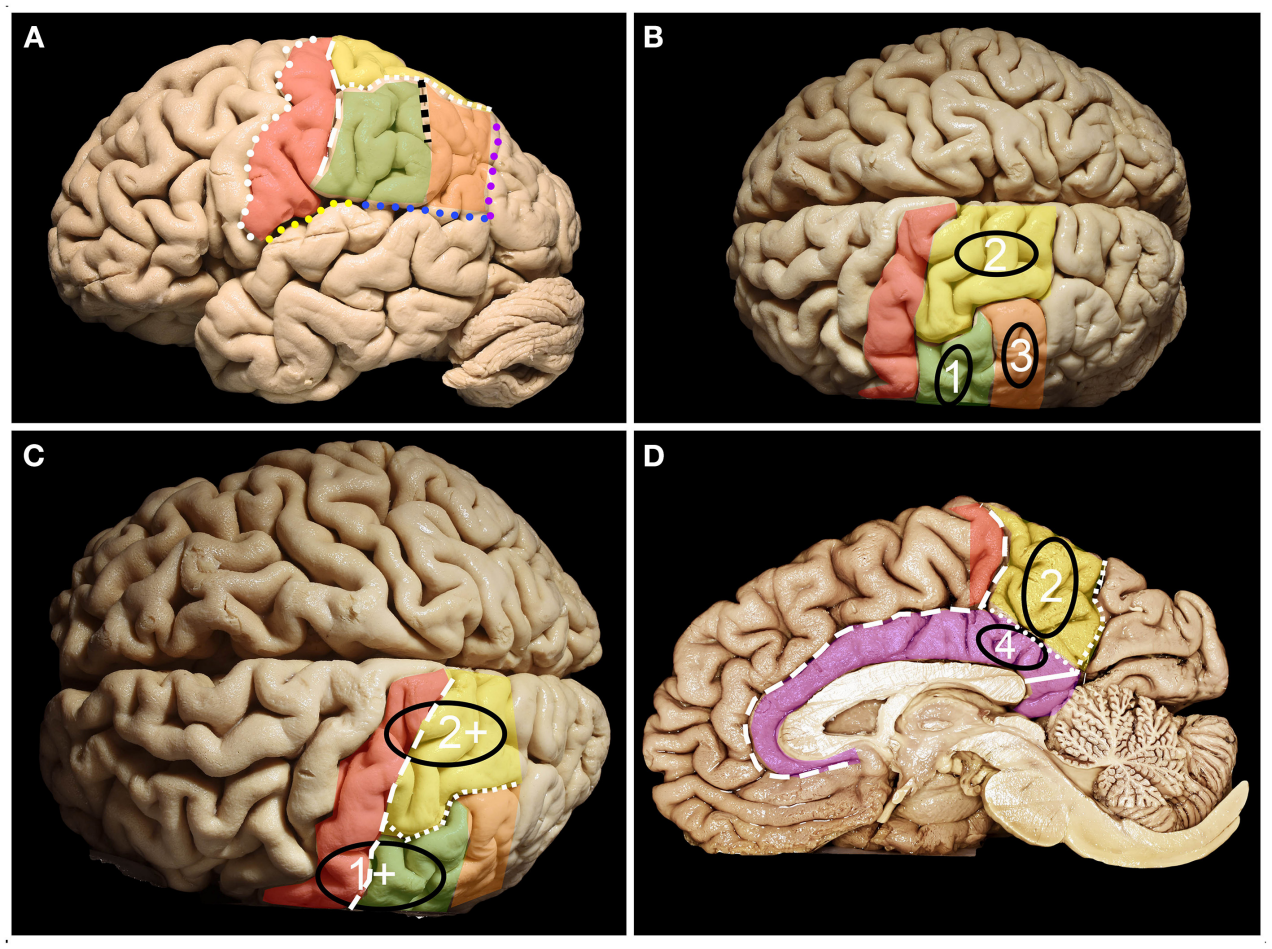
Anatomy of the parietal lobe from lateral, mesial and superior perspectives. **(A)** The parietal lobe is placed posteriorly to the central sulcus (white dots), which is one of the most constant landmarks on the convexity of the brain. The posterior border and inferior border of the parietal lobe on the lateral surface are marked by the temporoparietal line (purple dots) and parieto-occipital line (blue dots) with the posterior ramus of the lateral sulcus (yellow dots), respectively. Running parallel to the central sulcus, the postcentral gyrus (red) can be identified, which is limited posteriorly by the posterior central sulcus (white rectangles). Posterior to the postcentral sulcus, superior (yellow) (SPL) and inferior (green + orange) parietal (IPL) lobules are identified, which are separated by the intraparietal sulcus (white squares) (IPS). The IPS and postcentral sulcus junction patterns can be defined according to the Cunningham into one of the five subtypes: I. postcentral sulcus is divided into superior and inferior portion, IPS separate; II. inferior post-central confluent with IPS, superior postcentral separate; III. superior and inferior post-central confluent, IPS separate; IV. superior and inferior postcentral confluent and unified with IPS; V. IPS confluent with the superior post-central and inferior post-central separate (4). The superior branch of the IPS (the sulcus of Brissaud) is localized within the SPL. The inferior branch of the IPS (black squares) (the sulcus of Jensen) separates the SMG (green) anteriorly from the AG (orange) posteriorly. **(B)** Parietal lobe glioma classification according to Berger et al. distinct three zones 1, 2 and 3 on the lateral brain surface that correspond to SMG, SPL and AG retrospectively. **(C)** Tumors in zones 1 and 2 extending anteriorly beyond the SMG and SPL become tumors within zones 1+ and 2+. **(D)** Zone 2 consists of the mesial surface of the precuneus, while Zone 4 consists of the gyrus cinguli below the precuneus. On the medial surface of the hemisphere. The parietal lobe is defined by the precuneus (yellow) and the posterior part of the paracentral lobule (red). The anterior border of the precuneus is marked by the marginal branch of the cingulate sulcus (white rectangles), posterior by the parieto-occipital sulcus (white squares) and inferior by the subparietal sulcus (white dots). Below which the cingulum (purple) covering the corpus callosum is identified. The isthmus of the cingulum (white continuous line) at the level of the posterior surface of the splenium of the corpus callosum was identified.

**Figure 2:**
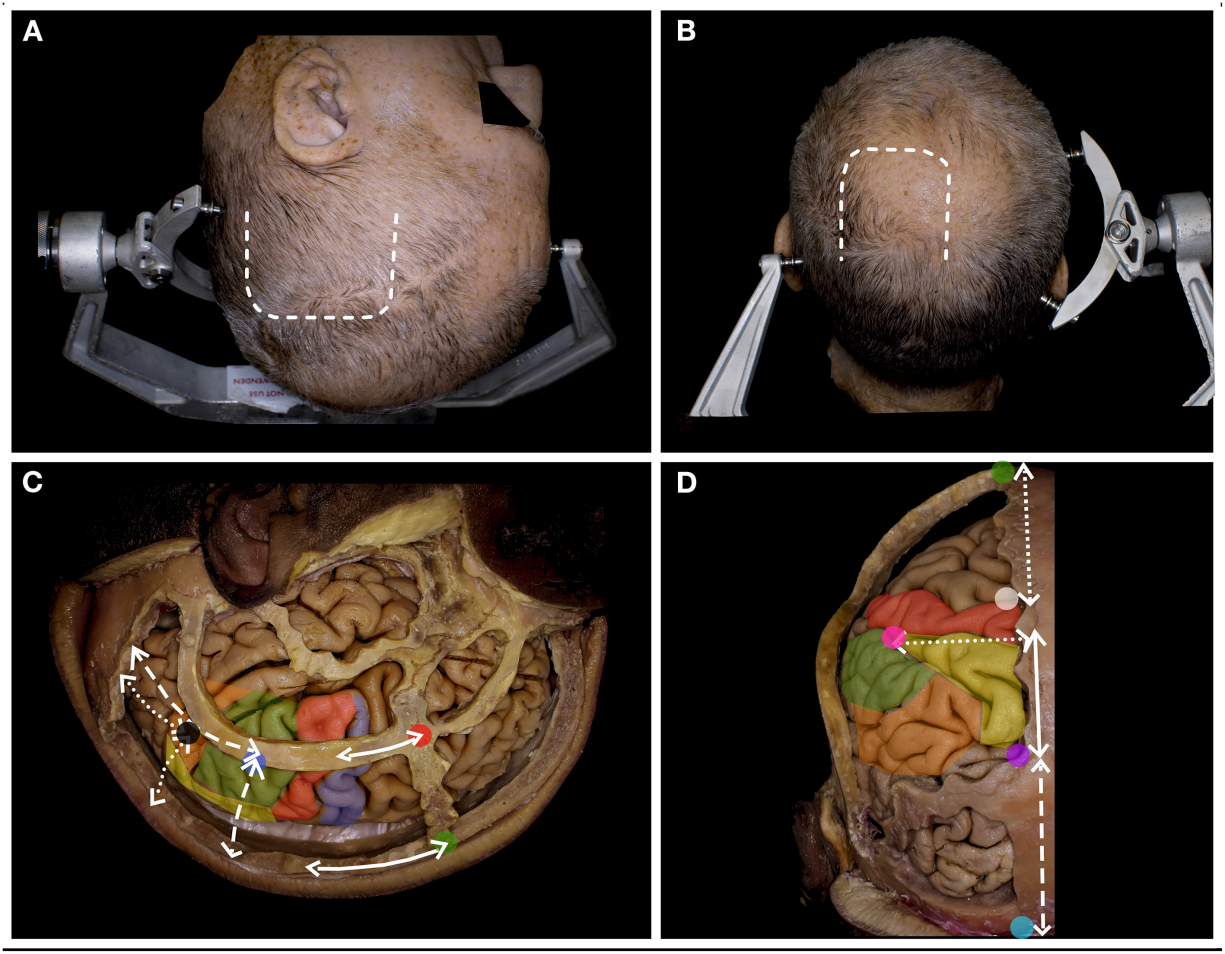
The surgical perspective of the parietal lobe anatomy. **(A)** For the tumors located within Zones 1, 1+ and 3, below the IPS, the lateral trajectory is preferred, and the long axis of the head is rotated to the contralateral side, being almost parallel to the floor. A u-shaped skin incision (white rectangle) based on the skull base is preferred. **(B)** For tumors located above the IPS within Zones 2, 2+, and 4, the superior trajectory is preferred, and the head is positioned in the neutral position. A u-shaped skin incision (white rectangle) with the base at the transverse sinus is preferred. **(C)** The lateral perspective of the parietal lobe in relation to the craniometric points with marked precentral gyrus (blue), the postcentral (red), the SPL (yellow), the SMG (green) and the AG (yellow). The postcentral gyrus (along the continuous white line) is located ~6.5 cm posterior to bregma (green dot) and 4.1 cm behind stephanion (red dot). The end of the lateral sulcus within the SMG (blue dot) is identified (along the white rectangle line) ~6.0 cm anterior to the lambdoid suture and 6.4 cm lateral to the sagittal suture. The end of the superior temporal sulcus within the AG (black dot) was identified (along the white dotted line) ~4.3 cm from the lambdoid and 5.0 cm from the sagittal suture (6). **(D)** The posterior/superior perspective of the parietal lobe in relation to the craniometric points. The superior Rolandic point (white dot) is identified ~5.0 cm behind bregma, and behind this point, the postcentral gyrus is identified. The parieto-occipital sulcus (purple dot), which marks the posterior border of the parietal lobe, is identified at the level where the lambdoid suture joins the sagittal suture. This point is ~6 cm anterior to the inion (light blue) along the midline (white rectangle line). Another 6.0 cm anterior to the lambdoid suture along the sagittal suture and 5.0 cm lateral and perpendicular to perpendicular to the midline the point (pink) where IPS joins the postcentral sulcus is identified (7).

**Figure 3:**
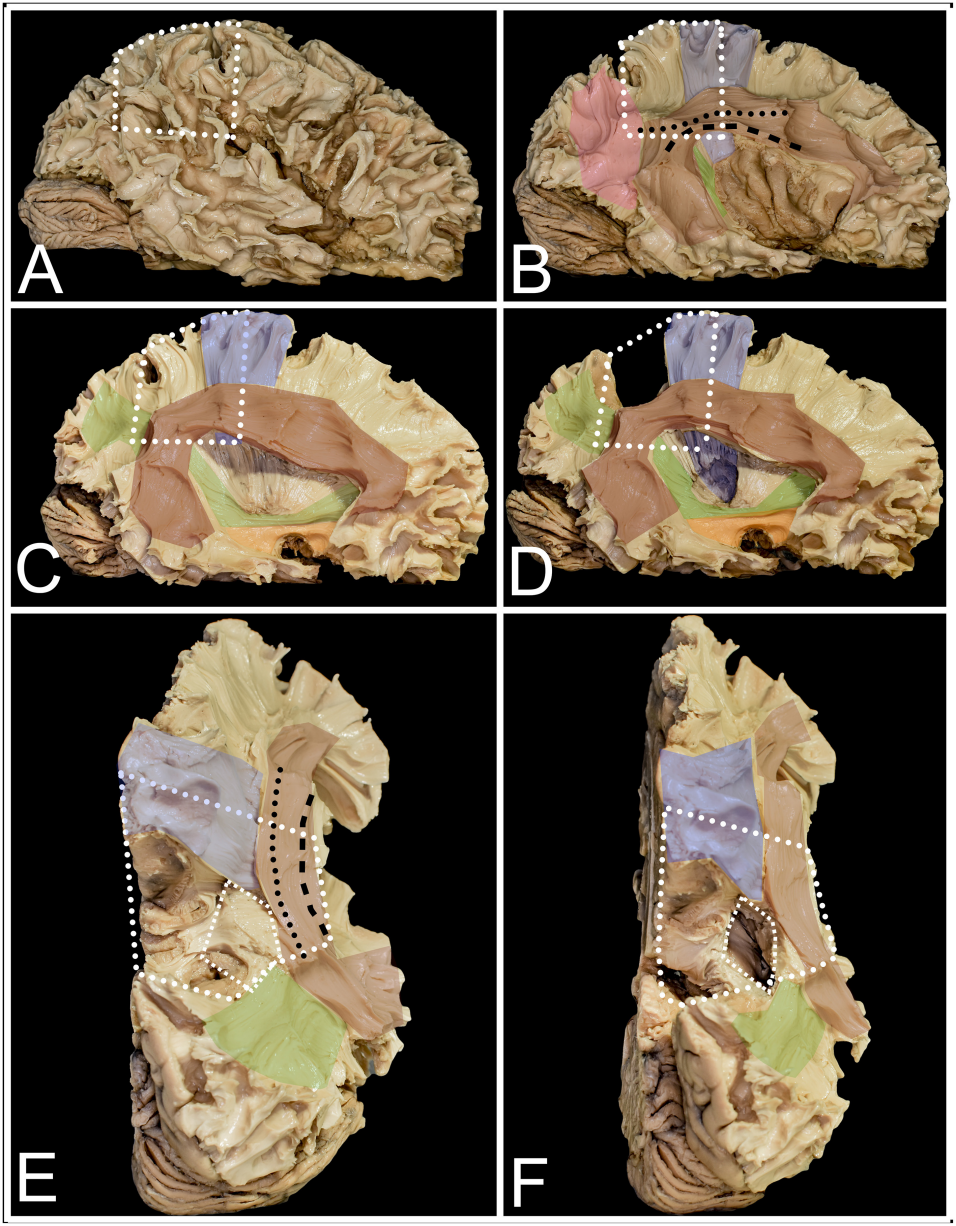
The figure presents the anatomy of white matter tracts within and outside the parietal lobe from lateral **(A–D)** and superior **(E,F)** perspectives. White dots mark the localization of the parietal lobe within the right hemisphere. **(A)** After removal of the cerebral cortex, the short u-fibers connecting neighboring gyri are exposed. **(B)** The most superficial layer of the associated fibers is formed by the AF/SLF complex (burgundy) and by the vertical occipital fasciculus (VOF) (pink). The most superficial layer of the AF/SLF complex is formed by SLF II (black dots), which connects the angular gyrus with the middle frontal gyrus. SLF III (black rectangle) runs inferior to SLF II, without a clear demarcation between them, connecting the SMG with the inferior frontal gyrus. Medial to SLFs II and III, the AF is identified, which is difficult to separate from the SLF complex, as they have the same direction on the horizontal segment of the AF/SLF complex. Under the vertical part of the AF/SLF complex in the anteroposterior direction, the fibers of the sagittal stratum (SS) (green) are exposed. The most superficial layer of the fibers within the SS is formed by the ILF, but they run inferior to the atrium and are not related to parietal lobe surgery. A deeper layer is formed by the IFOF, which covers the whole lateral wall of the atrium. The deepest segment is formed by optic radiation (OR), which at the bottom is fused with the anterior commissure (AC). Deeper to the OR and directed perpendicular to it, the fibers of the tapetum of the lateral ventricle were identified. Mesial to the AF/SLF complex at the level of the paracentral lobule, corticospinal tract (blue) fibers are identified. **(C)** The mean compartment of the SS is formed by the IFOF, which is identified when the cortex of the insula is removed. Ventral to the IFOF, the uncinate fasciculus (UF) (orange), which connects the frontal and temporal lobes, is identified. **(D)** The precuneus was resected to expose the fibers of the paracentral lobule that marked the anterior margin of the resection. The posterior part is formed from thalamocortical fibers, which are sensory fibers. Below the AF/SLF complex, the corticospinal tract forms the posterior limb of the internal capsule, which is covered laterally by the putamen. **(E)** The superior perspective after removal of the precuneus. The surface marked with a line of white squares corresponds to the position of the atrium of the lateral ventricle. **(F)** The atrium of the lateral ventricle was opened from the superior ventricle. Lateral to it, the horizontal segment of the AF/SLF complex is identified, and posteriorly, the superior fibers form the SS.

The parietal lobe is the third most common site of glioma incidence in adult patients, following the frontal and temporal lobes (1). In addition to containing cortical regions related to sensory and language function at the subcortical level, the parietal lobe is a crossroads of white matter tracts related to motor, sensory, language, visuospatial, and visual function (2). However, in a study of a large cohort of patients operated on due to parietal lobe gliomas, characteristic parietal lobe syndromes (neglect syndromes; visuospatial dysfunction; Gerstman's syndrome – agraphia4, acalculia, finger agnosia) were infrequently observed in the long term (3). In contrast to the characteristic parietal lobe syndromes, language and visual field deficits are frequently associated with surgery on the parietal lobe, occurring significantly more often with such procedures than with surgery in other sites, and these deficits negatively impact patients' quality of life. Surgical approaches to the parietal lobe are mainly based on the lesion's localization in relation to the ventricular system and the main white matter tracts of the sagittal stratum (SS) and arcuate fasciculus/superior longitudinal fasciculus complex (AF/SLF complex) (Figure 1). From an anatomical perspective, the parietal lobe has four main components: the postcentral gyrus, the inferior parietal lobule (IPL), the superior parietal lobule (SPL), and, on the medial surface, the precuneus, which merges with the SPL on the superior margin of the hemisphere (5) (Figures 1, 2). From a neurosurgical perspective, six different types of tumor infiltration within the parietal lobe have been identified based on the classification proposed by Sanai et al. (3) (Figure 2). In the surgical classification, the posterior part of the cingulum, which is anatomically part of the limbic system, is incorporated into the parietal lobe. Tumors within Zones 1 and 3, which correspond to the supramarginal gyrus (SMG) and angular gyrus (AG), are highly related to permanent postoperative language and visual deficits, respectively. In Zones 2 and 4, extensive surgical resections are possible due to a low risk of permanent neurocognitive deficits and are mainly limited anteriorly by the corticospinal and thalamocortical tracts (Figure 3). Tumor localization is important in terms of anticipating the risk of the procedure as well as selecting the type of intraoperative mapping and the surgical approach. We studied in detail the cortical anatomy of the parietal lobe as well as of white matter tracts and its relationship to the brain surface, which must be taken into consideration during surgical procedures.

## Materials and Methods

Five adult human cadaveric brains were prepared following the Klingler technique; the details of the technique have been described previously (8). Briefly, the specimens were fixed with 4% formalin for at least 4 weeks, and the arachnoid and vessels were removed; afterwards, each brain was stored dry in a freezer at −15 degrees Celsius for 2 weeks. For thawing and preservation, 4% formalin solution at room temperature was used. The brain was placed in a position simulating an intraoperative scenario. For Zones 2, 2+, and 4, the long axis of the brain was perpendicular (to present perspective from superior trajectory) or parallel (to present perspective from lateral trajectory) to the floor for Zones 1, 1+, and 3 (Figure 2). Measurements were made with an electric digital caliper, protractor and measuring tape. Cortical anatomy was assessed with a naked eye and all measurements on the brain surface were performed with a measuring tape and protractor. In the next step, white matter dissection was performed with the aid of surgical microscope. All measurements on the subcortical level were performed with the electric digital caliper and protractor. Each measurement was taken twice by the same observer and the average value was taken for presentation of the results. A digital camera (Nikon D7200 with a Nikon DX 35 mm 1:1.8 G lens) was used for image documentation. The study was approved by the Bioethics Committee of Medical University of Warsaw, approval number AKBE/126/2019.

## Results

### Lateral Approach

#### Measurements on the Cortical Level

**Figure 4:**
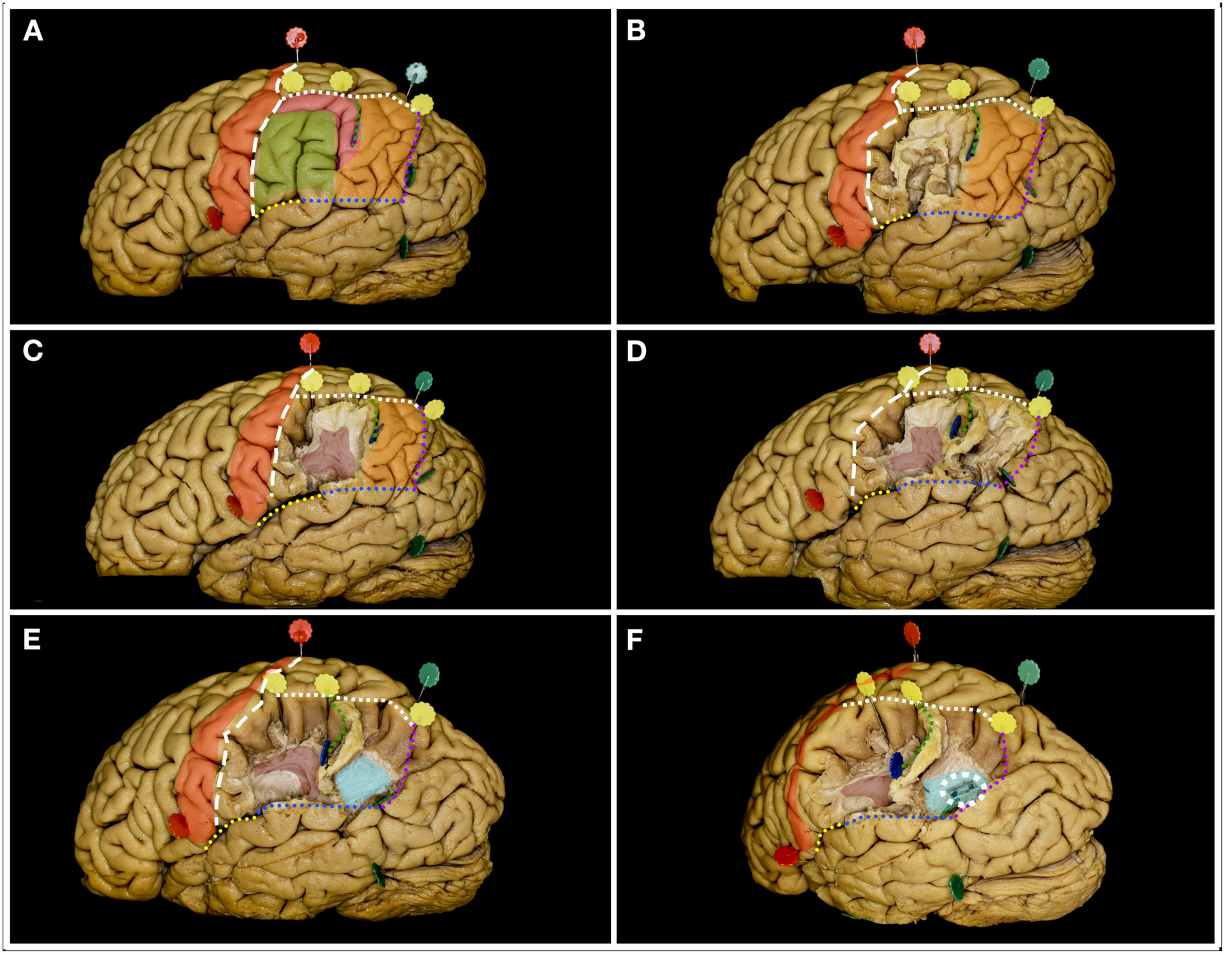
The figure represents the surgical perspective of the surgical field within the inferior parietal lobule from a lateral perspective. **(A)** The IPL is located posterior to the postcentral sulcus (white rectangle line) and to the postcentral gyrus (red), inferior to the intraparietal sulcus (white square line), anterior to the parietotemporal line (purple dots) and superior to the occipitotemporal line (blue dots) posteriorly and the end anteriorly by the posterior ramus of the lateral fissure (yellow dots). Within the IPL, the SMG (green) and AG (orange) are identified. In the typical pattern no additional gyri behind the postcentral sulcus within the IPL can be identified. When additional gyrus is located between the postcentral sulcus and the SMG or between SMG and AG the pattern is defined as PreSMG or PreAG, retrospectively (9). In this case, the additional gyrus (pink) was identified between the AG and SMG, bordering the anterior edge of the AG. The two gyri are separated by the inferior ramus of the IPS, the sulcus of Jensen (green square line). **(B)** Here, the most superficial layer of the fibers within the SMG was constituted by short u-fibers. **(C)** Removal of the u-fiber system reveals the AF/SLF system (burgundy), its genu and posterior segment of the horizontal as well as superior segment of the vertical ramus. The sulcus of Jensen marks the point posterior to the most posterior point of the genu of the AF/SLF complex. **(D)** Posteriorly within the AG, the u-fibers go deeper than anteriorly. **(E)** After removal of these fibers, fibers deeper than the AF/SLF complex within the SMG, the fibers of the sagittal stratum (light blue) running in the anteroposterior direction were identified. **(F)** When the SS fibers were separated, the occipital horn and posterior part of the atrium of the lateral ventricle (white square circle) were identified.

The postcentral sulcus, which marks the anterior margin of the IPL, was identified 18.1 (ranging from 14 to 22) mm posterior to the inferior Rolandic point (IRP) (Figure 4). The inferior border was formed by the occipitotemporal line, which had 41.4 (ranging from 33 to 49) mm. The superior border was formed by the intraparietal sulcus (IPS), which was connected to the postcentral sulcus in all cases. The pattern of confluence of the IPS with the postcentral sulcus was as follows: I (0), II (3), III (0), IV (10), V (1) (descriptions of different patterns of confluence of the IPS with the postcentral sulcus are described in Figure 1). The point where the IPS joins the postcentral sulcus was located 40.5 (ranging from 33 to 50) mm and 36.7 (ranging from 28 to 46) mm from the interhemispheric fissure (IHF) and lateral sulcus, respectively. The mean angle where the IPS joined the postcentral sulcus was 114 (ranging from 96 to 155) degrees. The length of the IPS varied from 22 to 47 (mean 36.5) mm. The long axis of the IPS was at 107.4 (varied from 180 - parallel to 110 degrees) angles to the IHF. The posterior end of the IPS was localized 27.4 (varied from 19 to 38) and 59.5 (varied from 48 to 67) mm from the IHF and preoccipital notch, respectively. The inferior branch of the IPS (sulcus of Jensen) was identified in 9/10 cases and measured from 9 to 45 (mean 26.7) mm, while the superior branch (sulcus of Brissaud) was also identified in 9 cases and measured 17.9 (ranging from 4 to 32) mm.

#### Measurements on the Subcortical Level

Within the SMG, the AF/SLF complex was identified; close to the sulcus of Jensen, the genu of the complex was the horizontal ramus joining the vertical ramus. This point was identified behind the postcentral sulcus at ~22.4 (ranging from 17 to 35) mm, anterior to the most posterior point of the IPS at ~24.2 (ranging from 12 to 35) mm at a depth of 22.6 (ranging from 21 to 24) mm from the cortical surface. Posterior to the sulcus of Jensen and in all cases posterior to the artificial line connecting the preoccipital notch with the SRP within the AG, the fibers of the SS at ~28.6 (ranging from 27 to 30) mm were identified. The SS was ~5 mm thick, and the lateral ventricle was identified at ~32.4 (ranging from 29 to 35) mm. The occipitotemporal line started 33.1 (ranging from 23 to 43) mm along the parietotemporal line and had an average of 44.4 (ranging from 32 to 75) mm. From its sylvian end, the IRP was identified in an additional 14.4 (varied from 8 to 23) mm anteriorly (Table 1).

**Table 1:** Measurements related to the lateral approach.

	**Average**	**Range**
**Superior approach**		
**Lateral surface**		
The inferior Rolandic point to the postcentral sulcus	18.1 mm	14–22 mm
Length of the occipitotemporal line	41.4 mm	33–39 mm
IPS-postcentral sulcus to the IHF	40.5 mm	33–50 mm
IPS-postcentral sulcus to the lateral sulcus	36.7 mm	28–46 mm
IPS-parietotemporal line to the IHF	27.4 mm	19–38 mm
IPS-parietotemporal line to the preoccipital notch	59.5 mm	48–67 mm
Length of the sulcus of Jensen	26.7 mm	9–45 mm
Length of the sulcus of Brissaud	17.9 mm	4–32 mm
**Subcortical level**		
Genu of the AF/SLF complex to the postcentral sulcus	22.4 mm	17–35 mm
Genu of the AF/SLF complex to the occipitotemporal line	24.2 mm	12–35 mm
Depth to the genu of the AF/SLF complex	22.6 mm	21–24 mm
Depth to the SS complex	28.6 mm	27–30 mm
Depth to the occipital horn	32.4 mm	29–35 mm

### Superior Approach

#### Measurements on the Lateral Surface of the Hemisphere

**Figure 5:**
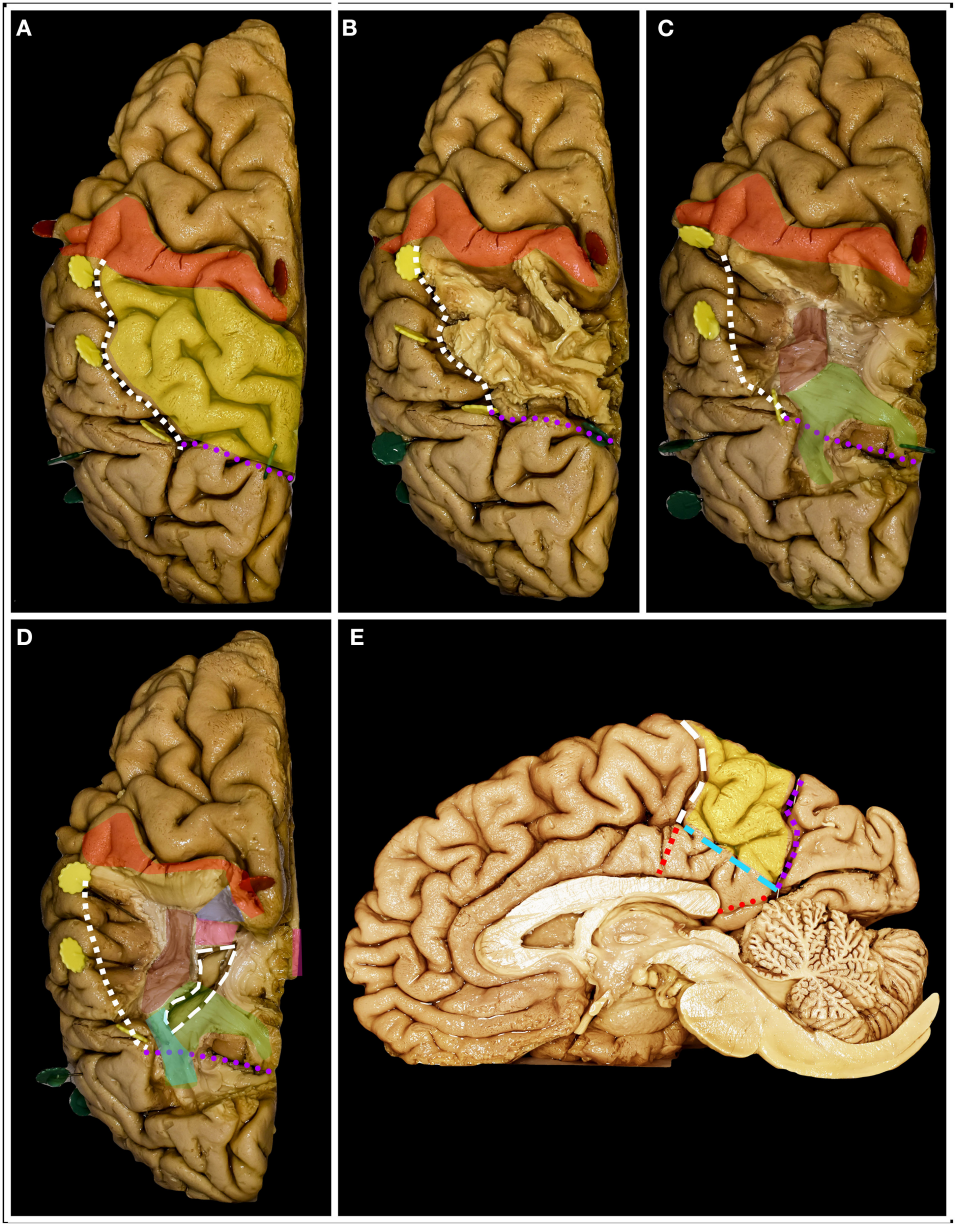
The figure represents the surgical perspective of the surgical field within the superior parietal lobule from a superior perspective. **(A)** The SPL is located posterior to the postcentral gyrus (red), mesial to the intraparietal sulcus (white square outline), anterior to the parietotemporal line (purple dots) and lateral to the interhemispheric fissure. **(B)** The most superficial system of the fibers is constituted by the u-fibers connecting the neighboring gyri going at the bottom of the fissures, which are quite deep in this region, reaching up to the gyrus cinguli. **(C)** Removal of the u-fibers laterally reveals the fibers of the AF/SLF complex (burgundy), which in this case were located mesial to the anterior part of the IPS. Posteriorly and deeper to the AF/SLF fibers of the corona radiata (green) is identified. **(D)** Mesial the most medial fibers of the AF/SLF complex, the atrium and the occipital horn (white rectangular outline) of the lateral ventricle are identified. The lateral ventricle was identified at the level of the corpus callosum (pink). Anteriorly at the level of the postcentral gyrus, the fibers going medial and deeper than the AF/SLF complex are the thalamocortical fibers (blue). Posterior to the vertical segment of the AF/SLF complex, on the lateral wall of the atrium and occipital horn of the lateral ventricle, the SS is identified. **(E)** On the mesial surface, the precuneus (yellow) constitutes the mesial part of the SPL. The resection line can be performed along the well-defined parieto-occipital (purple square outline) sulcus posteriorly and marginal ramus of the cingulate sulcus (white rectangular outline) anteriorly. The anatomical inferior border of the resection is set by the subparietal sulcus (light blue rectangle line) or surgical sulcus on the corpus callosum along the line being extension of the marginal ramus (red square line) and of the calcarine sulcus through the isthmus (red dots).

The superior Rolandic point (SRP) and the parieto-occipital sulcus mark the superior and inferior borders of the superior parietal lobule on the midline (Figure 5). The SRP was located 134.9 (ranging from 125 to 140) mm and 104.8 (ranging from 83 to 128) mm from the frontal and occipital bases along the superior margin of the hemisphere, respectively. The long axis of the central sulcus was identified at 109.1 (ranging from 95 to 118) degrees with the IHF and had a length of 85.8 (ranging from 77 to 94) mm. The postcentral sulcus, which defined the posterior border of the primary sensory cortex, was identified 11.2 (ranging from 4 to 23) mm posterior to the SRP. The distances from the SRP and from the postcentral sulcus to the parieto-occipital sulcus were 45.8 (ranging from 36 to 57) mm and 38.7 (ranging from 24 to 47) mm, respectively. The parietotemporal line marks the posterior border of the SPL and its long axis were identified at 73.8 (ranging from 65 to 82) degrees angle to the IHF and had a length of 77.3 (ranging from 70 to 88) mm.

#### Measurement on the Medial Surface of the Hemisphere

The superior margin of the precuneus had a length of 37 (varying from 31 to 43) mm, while the posterior part of the paracentral lobule was 10.4 (ranging from 4 to 22) mm, and the paracentral lobule was 27.3 (ranging from 19 to 38) mm. The posterior border of the SPL is formed on the medial surface by the parieto-occipital sulcus, which is 24.2 (ranging from 14 to 32) mm and was identified at a 69.2 (ranging from 60 to 85) degree angle to the superior margin of the hemisphere. The marginal branch of the cingulate sulcus stating for the anterior border of the SPL had a length of 21.5 (ranging from 18 to 28) mm and was identified at 99 (ranging from 75 to 130) degrees to the superior margin of the hemisphere. The line connecting the cingulate-marginal sulcus connection point with the parieto-occipital-calcarine connection point (subparietal sulcus), which corresponds to the anatomical inferior border of the SPL on the mesial surface of the hemisphere, had a length of 37.1 (ranging from 32 to 40) mm. The width of the cingulate gyrus below the marginal sulcus was 16.3 (ranging from 11 to 21) mm in the line being extension of the marginal sulcus. Along the same trajectory, the width of the corpus callosum was 6.3 (ranging from 4 to 8) mm. The parieto-occipital-calcarine connection point was located 22 (ranging from 19 to 28) mm posterior to the splenium of the corpus callosum, along the calcarine sulcus through the isthmus.

#### Measurement on the Subcortical Level

From the lateral perspective, from the superior perspective, the main white matter tracts that are taken into consideration during the procedure are thalamocortical/corticospinal tract anteriorly; AF/SLF complex located lateral and anteriorly; SS located in line with the horizontal segment and behind the previous segment. The most mesial fibers of the AF/SLF complex were identified ~33.2 (ranging from 27 to 40) mm lateral to the IHF and ~25.6 (ranging from 23 to 28) mm from the cortical surface. In 60% of patients, the IPS was in line with the horizontal segment of the AF/SLF complex; in rest, its anterior point was located lateral to the genu of the AF/SLF complex. The occipital horn of the lateral ventricle was identified ~9 (ranging from 2 to 16) mm mesial to the AF/SLF complex at ~34 (ranging from 32 to 36) mm, measured perpendicular to the brain surface. Resection ended on the inferior anatomical border of the parietal lobe; at the level of the subparietal sulcus, the lateral ventricle should not be encountered (Table 2).

**Table 2:** Measurements related to the superior approach.

	**Average**	**Range**
**Superior approach**		
**Lateral surface**		
The superior Rolandic point to frontal base	134.9 mm	125–140 mm
The superior Rolandic point to occipital base	104.8 mm	83–128 mm
The superior Rolandic point to the parieto-occipital sulcus	45.8 mm	36–57 mm
The superior Rolandic point to postcentral sulcus	11.2 mm	4–23 mm
The long axis of the central sulcus to IHF	109.1 degrees	95–118 degrees
Length of the central sulcus	85.8 mm	77–94 mm
The long axis of the parietotemporal line to IHF	73.8 degrees	65–82 degrees
Length of the parietotemporal line	77.3 mm	70–80 mm
**Medial surface**		
Width of the paracentral lobule	27.3 mm	19–38 mm
The parieto-occipital sulcus to IHF	69.2 degrees	60–85 degrees
Length of the parieto-occipital sulcus	24.2 mm	14–32 mm
Length of the marginal branch of the cingulate sulcus	21.5 mm	18–28 mm
The marginal branch of the cingulate sulcus to the IHF	99 degrees	75–130 degrees
Length of the subparietal sulcus	37.1 mm	32–40 mm
**Subcortical level**		
AF/SLF complex from IHF (lateral)	33.2 mm	27–40 mm
Depth to the AF/SLF complex from cortical surface	25.6 mm	23–28 mm
Occipital horn to the mesial surface of the AF/SLF complex	9.0 mm	2–16 mm
Depth to the occipital horn from the cortex	34.0 mm	32–36 mm

## Illustrative Cases

### Case 1

**Figure 6:**
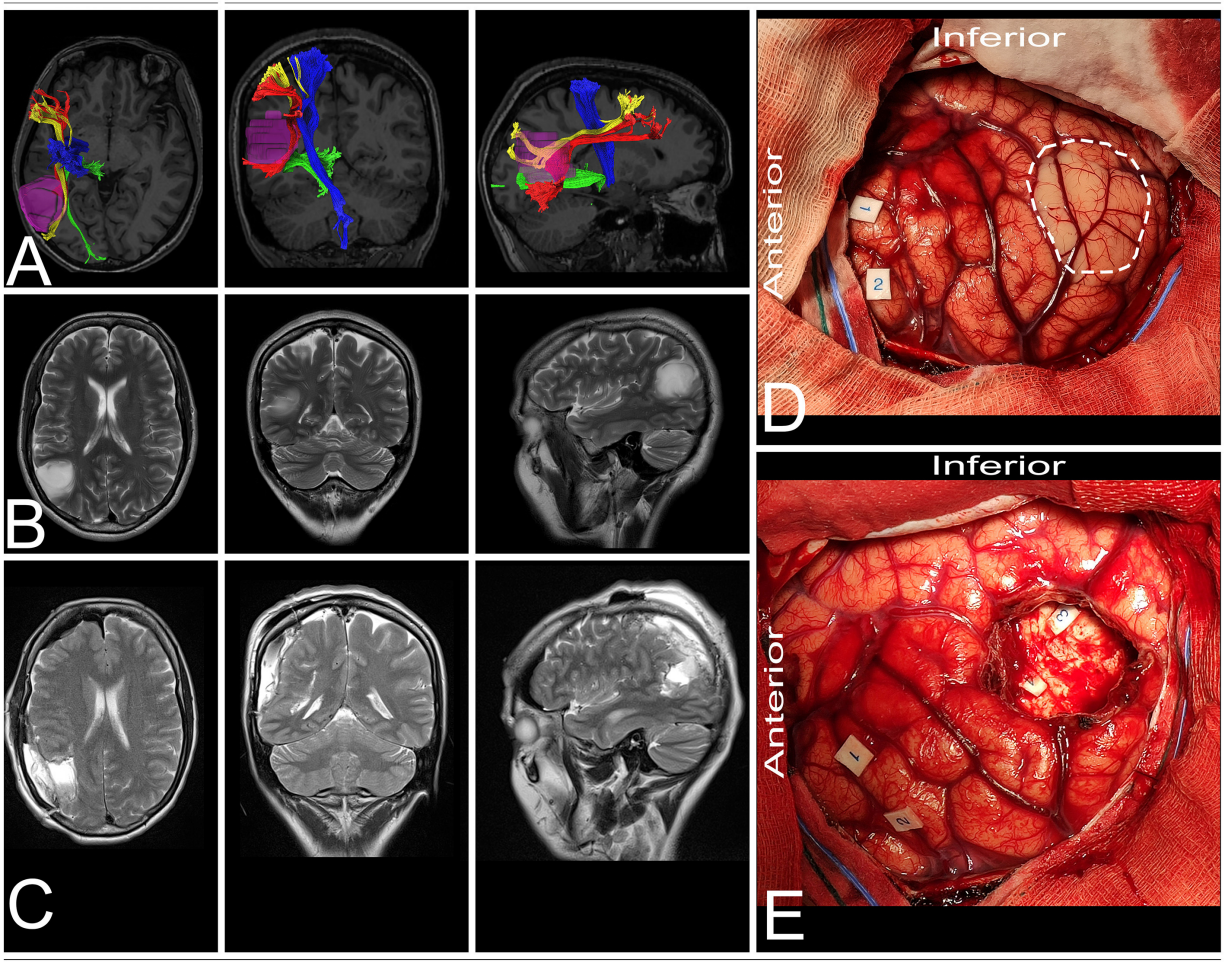
41-year-old right-handed women with a low-grade glioma within the non-dominant parietal lobe. **(A)** axial, coronal, and sagittal MRIs with diffusion tensor imaging tractography reconstruction of the tracts passing within the parietal lobe. The tumor (purple) is lateral to the AF (red), SLF II (yellow) and optic radiation (green), while posterior to the corticospinal tract (blue). **(B,C)** axial, coronal, and sagittal MRIs with preoperative and postoperative imaging. **(D)** Intraoperative photograph taken before tumor removal, after brain mapping. The tumor (white rectangle) was located posterior to the primary motor (tag 1 and 2) and sensory cortex. **(E)** Intraoperative photograph taken after tumor resection within functional borders, depth of resection is marked with optic radiation (tag 3) and SLF II (tag 4). Patient was operated in the lateral position with the head rotated to the left. DTI were reconstructed in DSI Studio (http://dsi-studio.labsolver.org) (11).

41-year-old right-handed women presented with a history of seizures without neurological deficit. MRI revealed a lesion characteristic of a low-grade glioma within the non-dominant parietal lobe, being lateral to the SS and AF/SLF complex (Figure 6). Patient was operated in the lateral position with the head rotated to the left. Intraoperative electrical stimulation of the cortex anterior to the tumor elicited responses from primary motor and sensory cortex within face and upper extremity. Stimulation of the white matter at the depth of resection elicited flashing within the left visual field and hemineglect syndrome when line bisection task was performed. These areas based on neuronavigation were related to optic radiation and SLF II, respectively. Resection was ended when eloquent structures were encountered. In the postoperative period patient presented mild hemineglect syndrome which improved in long term follow-up. The final neuropathology was WHO grade II astrocytoma.

### Case 2

**Figure 7:**
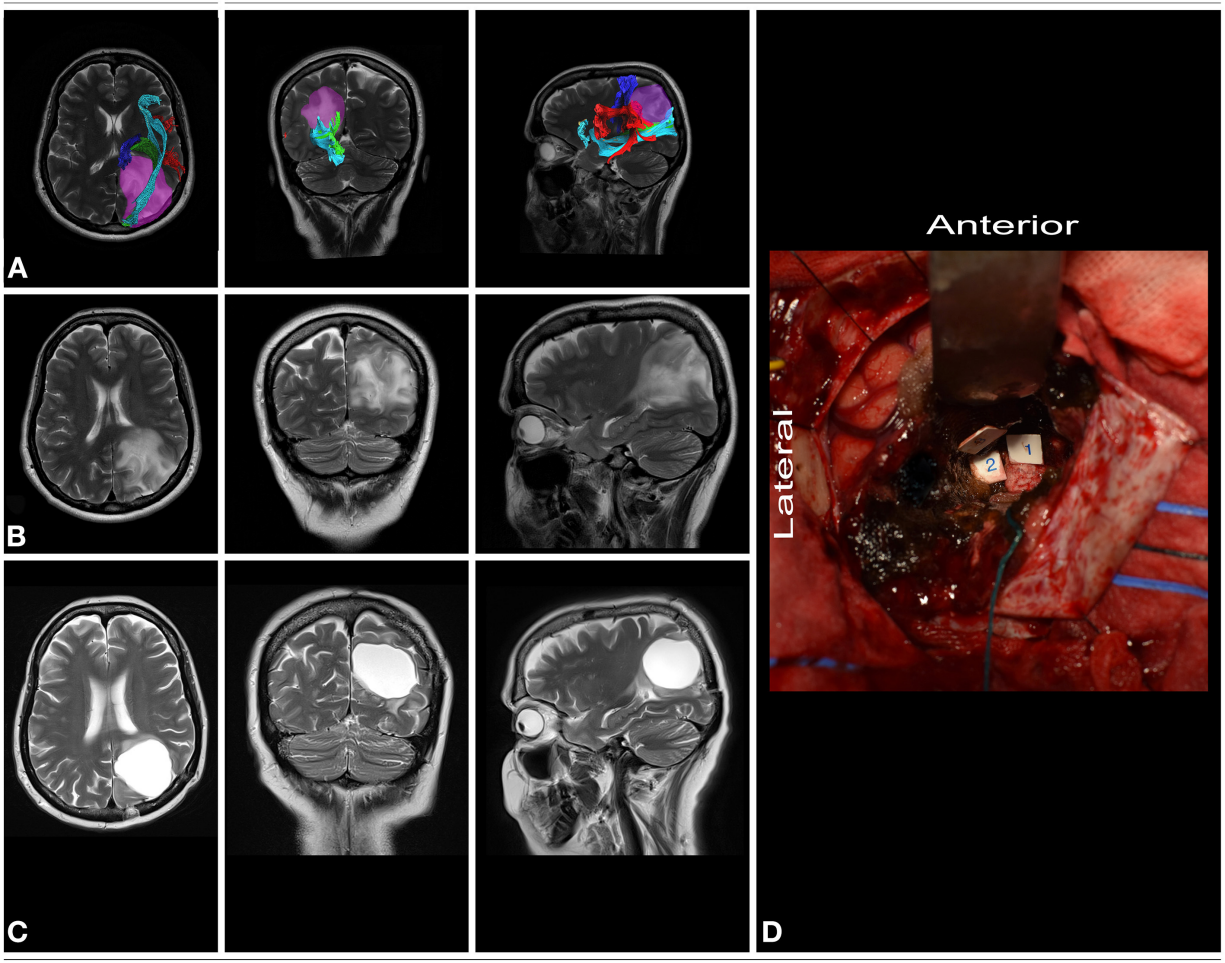
51-year-old right-handed women with a low-grade glioma within dominant parietal lobe. **(A)** axial, coronal, and sagittal MRIs with diffusion tensor imaging tractography reconstruction of the tracts passing within the parietal lobe. The tumor (purple) is medial to the AF/SLF complex (red), optic radiation (green) and IFOF (light blue), while posterior to the corticospinal tract (blue). **(B,C)** axial, coronal, and sagittal MRIs with preoperative and postoperative imaging. **(D)** Intraoperative photograph taken after tumor resection within functional borders, depth of resection is marked with corticospinal tract (tag 1), AF/SLF complex (tag 3) and the SS (tag 2). Patient was operated on in the semi-sitting position. DTI were reconstructed in DSI Studio (http://dsi-studio.labsolver.org) (11).

51-year-old right-handed women presented with a history of seizures without neurological deficit. MRI revealed a lesion characteristic of a low-grade glioma within the dominant parietal lobe, being medial to the SS and AF/SLF complex (Figure 7). Patient was operated in the semi-sitting position with the head in the neutral position. Intraoperative electrical stimulation of the cortex anterior to the tumor elicited responses from primary sensory and motor cortex. Stimulation of the white matter at depth of resection elicited movement disturbances on the anterior border of resection and flashing with language disturbances on the lateral/inferior border of resection. These areas based on neuronavigation were related to corticospinal tract, SS and AF/SLF complex, respectively. Resection was ended when eloquent structures were encountered. In the postoperative period patient presented with aphasia which improved in long term follow-up. The final neuropathology was WHO grade II astrocytoma.

## Discussion

Cortical and subcortical regions within the parietal lobe are related to core functions such as motor (proprioceptive disturbance), semantic (reading, writing and phonological processing), visuospatial function, and visual fields (2, 10). All of the above factors, except the visual field, cannot be assessed in an unconscious patient; therefore, the functional limits of resection can be identified only based on awake intraoperative brain mapping of the dominant and non-dominant hemispheres (12–15). In terms of the surgical approach to intra-axial lesions, we can distinguish those located medially (Zones 2 and 4) and laterally (Zones 1 and 3) in relation to the IPS and the atrium of the lateral ventricle on the cortical and subcortical levels, respectively. Surgical treatment of tumors within the inferior parietal lobule is related mainly to language and visual field deficits when located within Zone 1 and Zone 3, respectively. The risk of language-related deficits with this tumor is high (8.4%) compared to other locations in proximity to language pathways (3). When the patient undergoing surgery has a tumor in Zone 2 or 4, the main concern is the risk of motor deficits related to motor or sensory impairment. For safe and optimal surgical treatment, the surgeon should be familiar not only with the anatomical aspect of the approach but also with the functional significance of the cortex and white matter tracts that are encountered, in terms of chances of neuronal reorganization at the cortical level and functional restoration (16).

Despite the trajectory of the approach, the anterior border of the resection is constituted by the primary motor cortex within the precentral gyrus and the thalamocortical tract (sensory) at the subcortical level. The SRP can be easily estimated by cranial markers and is identified ~3.5–4.5 cm behind the coronal suture (5). Posterior to the motor cortex, the primary somatosensory cortex is identified and is marked posteriorly by the postcentral sulcus, which is identified ~1 and 2 cm posterior to the SRP and IRP, respectively, and runs almost parallel to the long axis of the central sulcus. Intraoperative stimulation of the primary somatosensory cortex and thalamocortical tracts under awake conditions produces different types of paresthesia, dysesthesia, or proprioceptive responses (17). Resection of the primary sensory cortex while preserving the cortex responsible for proprioception and the thalamocortical tract results in immediate postoperative sensory deficits, which will resolve in most cases due to plasticity and recruitment of the secondary somatosensory cortex, posterior parietal cortex, precentral cortex, or contralateral primary somatosensory cortex (2, 18, 19). Only awake conditions allow for an informed decision regarding the end of functional resection on the anterior border at the subcortical level. From an anatomical point of view, the posterior border of the parietal lobe is marked by the parietotemporal line, which measures ~8 cm and is located at ~70 degrees to the IHF. The posterior border of the resection despite the operated hemisphere is marked by the tumor, as long as it is above the calcarine sulcus, where the visual cortex can be assessed. The anatomical inferior border of the parietal lobe is constituted by the occipitotemporal line, which measures ~4 cm and is located ~8 cm lateral to the IHF. In the dominant hemisphere, the inferior border of the resection due to highly eloquent function should be based on intraoperative brain mapping (3, 12, 20). Wernicke's language area is located mainly within the posterior segment of the STG and SMG and occasionally posteriorly within the AG. These two gyri are separated by the sulcus of Jensen, which is perpendicular to the IPS and has a mean length of 2.5 cm. In some cases, atypical patterns in terms of additional gyri within the IPL should be expected. Mapping of the SMG is performed mainly with the use of picture-naming, and reading tasks are used. Positive language sites are identified when anomia, alexia or phonemic paraphasias are observed (2, 14). To avoid Gerstman syndrome, which is typically related to AG, finger recognition, writing, and calculation tasks are applied in mapping the posterior region of the IPL (2, 21). The resection above the IPS is most commonly negative to mapping, but intraoperative damage of the SPL may result in apraxic dysgraphia (22). One should keep in mind that the IPS, according to our observation, runs parallel to the IHF in only 40% of cases. The anterior end of the IPS was localized ~4 cm, while the posterior 2.5 cm lateral to the IHF and its long axis formed with the IHF at an angle of ~115 degrees. This may lead to the wrong assumption that when IPS is identified, the lesion mesial to the IPS is safely resectable. At the subcortical level, the IPS is linked to the AF/SLF complex as well as to the SS complex. From the lateral trajectory within the SMG, the depth border of the resection is marked by the AF/SLF complex, and its horizontal segment transitions into a vertical segment. From a lateral perspective, the complex is identified at a depth of ~25 mm from the cortical surface. On the dominant side, the main function of the SLF/AF complex is related to language, while on the non-dominant side, it is mainly due to visuospatial function. Intraoperative stimulation of the dominant AF/SLF complex results in expressive aphasia, phonemic paraphasias and repetition disorders (AF) or speech articulation dysfunction (SLF III), which are assessed during the picture naming task. On the non-dominant hemisphere, stimulation of the AF/SLF complex may result in contralateral hemineglect syndrome, which is related to the stimulation of the second compartment of the SLF (SLF II) (23), which is assessed with an intraoperative line bisection task (2, 24). The sagittal stratum is the most superficial fiber system, under the u-fibers posterior to the sulcus of Jensen or posterior half of the IPL. The SS complex is identified at ~30 mm. Deep to the SS, the lateral ventricle is identified, where distinction of the tracts forming it can be based only on intraoperative mapping. Transgression of the SS most commonly does not cause permanent neurological deficits except visual field deficits. The deep limit of the resection is represented by the visual pathway. Stimulation of the visual pathway may result in flashing, shadowing, or other unexpected visual experiences (25). For optic radiation, a modified picture naming task placed in quadrants is preferable. Laterally to the optic radiation the IFOF, responsible for semantic language processing in the dominant hemisphere with two subcomponents can be identified (26–28). Intraoperative stimulation of the dorsal and superficial subcomponent of the fibers that course in the superior portion of the SS and terminate within the superior parietal lobule may result in disturbances during reading and writing tasks (10, 26, 29). The second subcomponent of the IFOF which connects the frontal lobe with the inferior occipital cortex and posterior temporal-basal regions is located ventrally and deeper to the previous one and has no connections with the parietal lobe. Therefore, if the language pathway is stimulated, naming disturbances will occur without any subjective visual complaint (13, 30). The region superior and medial to the roof of the atrium is free of optic radiation and tracts of the sagittal stratum. The roof of the atrium can be reached through the dissection of the IPS perpendicular to the brain surface, and it marks the lateral border of the superior parietal lobule. The depth of the anterior half of the IPS is located ~1.25 cm above the atrium, while its posterior half is located above the occipital horn (31). The roof of the ventricular system corresponds to the level of the superior margin of the corpus callosum, and in this case, the trajectory of the dissection should be changed for the midline at the level of the roof of the ventricle. This allows preservation of the optic radiation, which courses on the lateral wall of the ventricle and avoids the eloquent cortical motor and speech areas that are located anteriorly and laterally to the planned corticotomy. Due to anatomical variability of the shape and localization of the IPS, the localization of white matter tracts must in each case be correlated with the sulcal and cortical anatomy. Based on our study and others, the AF/SLF complex might be identified mesial to the IPS, especially on the anterior segment of the IPS, which is why it is safer to start dissection in the middle of the length of the IPS (31). Resection of the mesial part of the parietal lobe also includes resection of the first segment of the SLF (SLF I), but the clinical impact of its damage is not well-described (32).

## Limitations of the Study

Readers should carefully consider our results, as we were not able to present all anatomical variants due to the limited number of specimens. The anatomical relationship between the structures in patients with gliomas is disturbed by the tumor mass, edema or due to brain shifts related to cerebrospinal fluid loss and tumor debulking. Additionally, the physical parameters of cadaveric brain specimen not exactly present an intraoperative brain structure. Despite the mentioned limitations, knowledge of physiological anatomy is of greatest importance even when the anatomy is disturbed.

## Data Availability Statement

The raw data supporting the conclusions of this article will be made available by the authors, without undue reservation.

## Ethics Statement

The studies involving human participants were reviewed and approved by Bioethics Committee of Medical University of Warsaw, approval number AKBE/126/2019. Written informed consent for participation was not required for this study in accordance with the national legislation and the institutional requirements.

## Author Contributions

TD, AB, and AM: conception/design of the work and final approval of the revision to be published. TD: data collection. TD and AM: data analysis and interpretation. TD and AB: drafting the article. AM: critical revision of the article. All authors contributed to the article and approved the submitted version.

## Conflict of Interest

The authors declare that the research was conducted in the absence of any commercial or financial relationships that could be construed as a potential conflict of interest.

## Publisher's Note

All claims expressed in this article are solely those of the authors and do not necessarily represent those of their affiliated organizations, or those of the publisher, the editors and the reviewers. Any product that may be evaluated in this article, or claim that may be made by its manufacturer, is not guaranteed or endorsed by the publisher.
